# Efficacy of liposomal irinotecan + 5-FU/LV vs. S-1 in gemcitabine-refractory metastatic pancreatic cancer: a real-world study using inverse probability of treatment weighting

**DOI:** 10.1007/s00535-024-02186-9

**Published:** 2024-11-30

**Authors:** Hiroshi Imaoka, Masafumi Ikeda, Satoshi Kobayashi, Akihiro Ohba, Masayuki Ueno, Yuko Suzuki, Hidetaka Tsumura, Nana Kimura, Shinya Kawaguchi, Yasuyuki Kawamoto, Kohei Nakachi, Kunihiro Tsuji, Noritoshi Kobayashi, Reiko Ashida, Naohiro Okano, Kumiko Umemoto, Gou Murohisa, Ayumu Hosokawa, Akinori Asagi, Hiroko Nebiki, Rei Suzuki, Takeshi Terashima, Ryusuke Shibata, Kazuhito Kawata, Toshifumi Doi, Hiroshi Ohyama, Yohei Kitano, Kazuhiko Shioji, Hiroyuki Okuyama, Atsushi Naganuma, Yuji Negoro, Yasunari Sakamoto, Satoshi Shimizu, Chigusa Morizane, Makoto Ueno, Junji Furuse, Hiroaki Nagano, the Japan Oncology Network in Hepatobiliary and Pancreas

**Affiliations:** 1 Department of Hepatobiliary and Pancreatic Oncology, National Cancer Center Hospital East, Kashiwa, Japan; 2 Department of Gastroenterology, Kanagawa Cancer Center, Yokohama, Japan; 3 Department of Hepatobiliary and Pancreatic Oncology, National Cancer Center Hospital, Tokyo, Japan; 4 Division of Gastrointestinal Oncology, Shizuoka Cancer Center, Shizuoka, Japan; 5 Department of Gastroenterology and Hepatology, Kurashiki Central Hospital, Kurashiki, Japan; 6 Department of Gastroenterology and Hepatology, Graduate School of Medicine, Kyoto University, Kyoto, Japan; 7 Department of Gastroenterology, Saitama Cancer Center, Saitama, Japan; 8 Department of Gastroenterological Oncology, Hyogo Cancer Center, Akashi, Japan; 9 Department of Surgery and Science, Faculty of Medicine, University of Toyama, Toyama, Japan; 10 Department of Gastroenterology, Shizuoka General Hospital, Shizuoka, Japan; 11 Division of Cancer Center, Hokkaido University Hospital, Sapporo, Japan; 12 Department of Medical Oncology, Tochigi Cancer Center, Utsunomiya, Japan; 13 Department of Gastroenterology, Ishikawa Prefectural Central Hospital, Kanazawa, Japan; 14 Gastroenterological Center, Yokohama City University Medical Center, Yokohama, Japan; 15 Second Department of Internal Medicine, Wakayama Medical University, Wakayama, Japan; 16 Department of Medical Oncology, Kyorin University Faculty of Medicine, Tokyo, Japan; 17 Department of Clinical Oncology, St. Marianna University School of Medicine, Kawasaki, Japan; 18 Department of Gastroenterology, Seirei Hamamatsu General Hospital, Hamamatsu, Japan; 19 Department of Clinical Oncology, University of Miyazaki Hospital, Miyazaki, Japan; 20 Department of Gastrointestinal Medical Oncology, National Hospital Organization Shikoku Cancer Center, Matsuyama, Japan; 21 Department of Gastroenterology, Osaka City General Hospital, Osaka, Japan; 22 Department of Gastroenterology, Fukushima Medical University, Fukushima, Japan; 23 Department of Gastroenterology, Kanazawa University Hospital, Kanazawa, Japan; 24 Digestive and Lifestyle Diseases, Kagoshima University Graduate School of Medical and Dental Sciences, Kagoshima, Japan; 25 Hepatology Division, Department of Internal Medicine II, Hamamatsu University School of Medicine, Hamamatsu, Japan; 26 Department of Molecular Gastroenterology and Hepatology, Kyoto Prefectural University of Medicine, Kyoto, Japan; 27 Department of Gastroenterology, Chiba University, Chiba, Japan; 28 Division of Gastroenterology, Department of Medicine, Asahikawa Medical University, Asahikawa, Japan; 29 Department of Internal Medicine, Niigata Cancer Center Hospital, Niigata, Japan; 30 Department of Medical Oncology, Kagawa University Hospital, Miki, Japan; 31 Department of Gastroenterology, National Hospital Organization Takasaki General Medical Center, Gunma, Japan; 32 Department of Oncological Medicine, Kochi Health Sciences Center, Kochi, Japan; 33 Department of Gastroenterology and Hepatology, International University of Health and Welfare Atami Hospital, Shizuoka, Japan; 34 Department of Gastroenterological, Breast and Endocrine Surgery, Yamaguchi University Graduate School of Medicine, Ube, Japan

**Keywords:** Pancreatic cancer, Second line, Liposomal irinotecan, S-1

## Abstract

**Background:**

S-1 monotherapy had previously been widely used as a second-line treatment for pancreatic cancer (PC) after gemcitabine-based chemotherapy mainly in Japan. Based on the results of the NAPOLI-1 trial, the recommended second-line therapy is now liposomal irinotecan plus fluorouracil/folinic acid (nal-IRI + 5-FU/LV). However, there have been no studies comparing nal-IRI + 5-FU/LV therapy with S-1 monotherapy.

**Methods:**

The main objective of this study was to compare overall survival (OS) in patients treated with nal-IRI + 5-FU/LV and those treated with S-1 monotherapy as second-line treatments, using the inverse probability of treatment weighting (IPTW) method. This study was conducted in 31 institutions participating in Japan Oncology Network in Hepatobiliary and Pancreas. To minimize potential biases due to the retrospective design, IPTW analysis was performed with multiple imputation, and imputed IPTW-adjusted hazard ratios and corresponding 95% confidence intervals (CIs) were estimated using a Cox proportional hazards model and combined into pooled estimates.

**Results:**

A total of 463 metastatic PC patients were enrolled in this study (257 in the S-1 monotherapy group and 206 in the nal-IRI + 5-FU/LV group). The median OS was 7.50 months (95% CI 4.18–12.69 months) in the nal-IRI + 5-FU/LV group and 5.72 months (95% CI 2.76–10.79 months) in the S-1 monotherapy group. In the IPTW-adjusted Cox proportional hazards model, nal-IRI + 5-FU/LV was associated with a significant OS benefit (pooled IPTW-adjusted hazard ratio, 0.779; 95% CI 0.399—0.941; *p* = 0.025).

**Conclusion:**

These findings support the use of nal-IRI + 5-FU/LV as standard second-line treatment for PC patients after gemcitabine-based chemotherapy.

**Supplementary Information:**

The online version contains supplementary material available at 10.1007/s00535-024-02186-9.

## Introduction

Pancreatic cancer (PC) is one of the deadliest cancers known and the seventh leading cause of cancer death. The numbers of newly diagnosed patients (510,566) and deaths (467,005) are almost the same because of its poor prognosis worldwide [1]. Approximately 60% of PC patients are diagnosed after metastasis has already occurred [2] [3] [4], and most patients are candidates for systemic chemotherapy. However, metastatic disease carries a dismal prognosis, with a 5-year survival rate of only 3%, which is the lowest known for any cancer type [5]. One possible reason for the poor prognosis is the fewer therapeutic options used to treat PC. Although the efficacy of combination chemotherapy for metastatic PC has been established in the first-line setting, most patients must discontinue these treatments due to disease progression or adverse events. For such patients, second-line treatment is generally attempted. However, there are fewer treatment options for second-line chemotherapy for PC, and the transition rate to second-line treatment for PC was reported to be only 40—60% [6] [7] [8]. Furthermore, in addition to PC’s aggressiveness, various complications (e.g., malnutrition, jaundice, and pain) lead to further challenges in providing chemotherapy to patients with PC. This trend is marked in the second-line setting.

Previously, the fluorouracil/folinic acid (5-FU/LV) or oxaliplatin in combination with 5-FU/LV regimen was used after gemcitabine-based chemotherapy, but the superiority of oxaliplatin in combination with 5-FU/LV regimen was controversial [9] [10]. However, the phase 3 NAPOLI-1 trial showed that the liposomal irinotecan plus 5-FU/LV (nal-IRI + 5-FU/LV) regimen significantly improved overall survival (OS) compared with the 5-FU/LV regimen [11], and now various guidelines recommend the nal-IRI + 5-FU/LV regimen as a second-line treatment for PC after gemcitabine-based treatment [12] [13] [14]. In contrast, in Japan, S-1 monotherapy had been widely used as a community standard for second-line treatment after gemcitabine-based treatment [15] [16] [17] until a phase 2 study demonstrated the efficacy of the nal-IRI + 5-FU/LV regimen in Japanese patients [18]. Currently, nal-IRI + 5-FU/LV therapy is recommended as second-line therapy for PC after gemcitabine-based treatment in Japan [19], but nal-IRI + 5-FU/LV therapy has not been compared with S-1 monotherapy.

S-1 is an oral fluoropyrimidine anticancer drug consisting of the fluorouracil prodrug tegafur combined with gimeracil and oteracil potassium. S-1 is convenient for patients and demonstrates efficacy and safety even in elderly patients [20]. Thus, S-1 monotherapy is still used as a second-line treatment option for PC [19]. Therefore, this multicenter, retrospective study (JON2109-P) was planned to compare the efficacy of nal-IRI + 5-FU/LV and S-1 monotherapy. The main objective of this study was to compare OS using the inverse probability of treatment weighting (IPTW) method in patients treated with nal-IRI + 5-FU/LV and those treated with S-1 monotherapy. The secondary objectives were to compare progression-free survival (PFS) using the IPTW method and tumor response in patients treated with nal-IRI + 5-FU/LV and those treated with S-1.

## Methods

### Study design

This study was conducted in 31 Japan Oncology Network in Hepatobiliary and Pancreas (JON-HBP) participating institutions in Japan. Patient data were collected using a secure, centralized database under the management of JON-HBP, and electronic case report forms were collected at the data center after eligibility had been assessed.

The inclusion criteria were as follows: histologically or cytologically confirmed adenocarcinoma; metastatic or recurrent PC patients (excluding recurrences occurring during adjuvant chemotherapy or within 6 months after the last adjuvant chemotherapy dose); initiation of either S-1 or nal-IRI + 5-FU/LV as the second-line treatment refractory to gemcitabine-based treatment between September 2019 and February 2021; age ≥ 20 years; and no prior exposure to irinotecan or fluoropyrimidine other than adjuvant chemotherapy. OS was measured from the date of start of second-line treatment to the date of death from any cause. OS for patients who were lost to follow-up was censored at the last date they were known to be alive. PFS was measured from the date of start of treatment to the date of first documented disease progression or the date of death from any cause. PFS was censored at the time of the last follow-up if there was no documentation of disease progression or death. Tumor response was based on the best overall response throughout the entire course of the observation period. Tumor responses were assessed using the Response Evaluation Criteria in Solid Tumors (RECIST) version 1.1.

This study was approved by the JON-HBP Protocol Review Committee and the institutional review board of National Cancer Center Hospital East. This trial was registered with the UMIN Clinical Trials Registry (https://www.umin.ac.jp/ctr/index-j.htm), number UMIN000048143. This study was performed in accordance with the international ethical recommendations of the Declaration of Helsinki and the Ethical Guidelines for Medical and Health Research Involving Human Subjects.

### Treatment

The standard S-1 monotherapy regimen is 80 mg/m^2^ of S-1 for 28 consecutive days, followed by a 14-day rest period, as described in the previous phase 3 trial [21]. The nal-IRI + 5-FU/LV regimen consisted of intravenous infusion of liposomal irinotecan 70 mg/m^2^ followed by folinic acid 400 mg/m^2^ with continuous fluorouracil 2,400 mg/m^2^ every 2 weeks, and the starting dose of liposomal irinotecan was reduced to 50 mg/m^2^ in patients with UGT1A1 *6 / *28 polymorphism, as described in the previous phase 3 trials [11]. Dose and schedule modifications were performed at the discretion of the treating physician.

### Statistical considerations

All patients who met the inclusion criteria at baseline were included as the full-cohort data, and their baseline characteristics in the nal-IRI + 5-FU/LV and S-1 groups were categorized and compared using the Chi-squared test. The time-to-event distribution was estimated using the Kaplan–Meier method, and the log-rank test was used to evaluate the differences between the nal-IRI + 5-FU/LV and S-1 groups in the full-cohort data.

This study has two potential biases due to its retrospective design. One is missing data, and the other is selection bias. Due to the retrospective design, covariate data were often missing. Missing data not only reduce the sample size, but may also lead to under- or over-estimation of treatment effects due to incomplete data. Selection bias, mainly caused by physician or patient choice, can affect the outcome of chemotherapy. To minimize these biases in this study, IPTW analysis with multiple imputation was performed.

When dealing with missing data, multiple imputation was performed under the assumption that the data were missing at random. Specifically, Rubin’s rules [22] were used to generate pooled effect estimates and variances across imputed datasets [23]. For multiple imputation, the logistic regression method was used for binary categorical covariates, and polytomous logistic regression methods were used for nominal categorical covariates. Five multiple imputation iterations were conducted, consistent with both the most commonly used guideline [22] and the most recent guidelines, which recommend conducting the same number of imputations as the average percentage rate of missingness for the data [24] [25]. Following multiple imputation, a complete-case analysis was also performed for survival outcomes using only cases with complete data, with no imputation, as a sensitivity analysis to validate the imputation model and evaluate the reliability of the results in accordance with the guideline [26].

In IPTW analysis, the weights are calculated as the inverse of the propensity score between the patient groups treated with nal-IRI + 5-FU/LV and with S-1. To evaluate the balance of covariates between the nal-IRI + 5-FU/LV and S-1 groups, standardized mean differences and variance ratios were used [27] [28]. In multiple imputation datasets, imputed IPTW-adjusted hazard ratios (HRs) and corresponding 95% confidence intervals (CIs) were calculated using a Cox proportional hazards model and combined into pooled estimates. In complete-case analysis datasets, IPTW-adjusted HRs and corresponding 95% CIs were calculated using a Cox proportional hazards model, and p values were calculated using an IPTW-adjusted log-rank test.

Exploratory subgroup analysis was performed to evaluate the interactions between baseline factors and treatment effect on OS; HRs for the nal-IRI + 5-FU/LV group vs. the S-1 group with their 95% CIs were estimated using a Cox proportional hazards model for each subgroup excluding patients with missing values.

Data were analyzed using STATA version 15.1 (StataCorp, College Station, TX, USA) and R version 4.3.2. (http://www.r-project.org/). The IPTW analysis and IPTW analysis with multiple imputations were performed using the WeightIt package version 0.14.2 [29] and MatchThem package version 1.1 in R [30], respectively. Covariate balance was assessed using Cobalt package version 4.5.1 in R [31]. *p* values < 0.05 were considered statistically significant, and all reported p values are two-sided. Details of the statistical methods and R codes for estimation of HRs for OS and PFS by IPTW analysis with multiple imputation are provided in Supplementary Appendix.

## Results

### Baseline characteristics

**Fig. 1:**
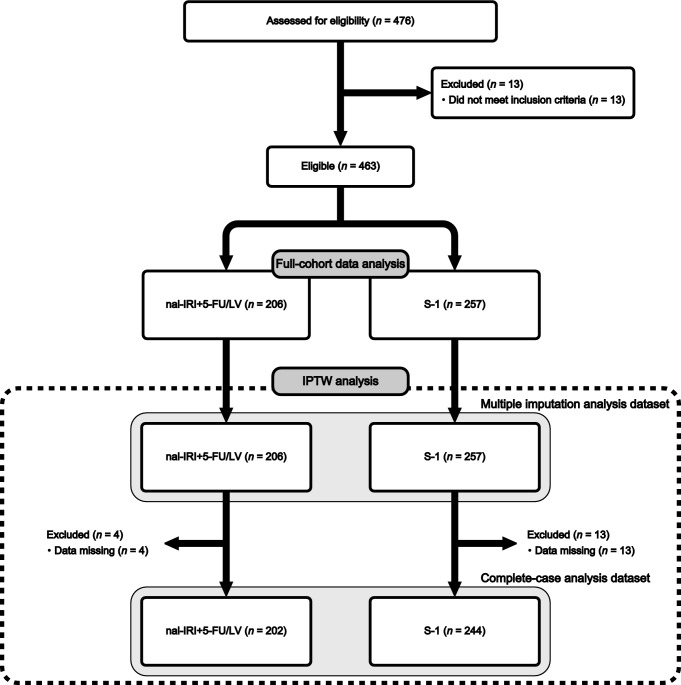
Patient flow chart. nal-IRI + 5-FU/LV, liposomal irinotecan plus fluorouracil/folinic acid

The patient flow chart is shown in Fig. 1. A total of 463 metastatic PC patients were enrolled in this study (206 in the nal-IRI + 5-FU/LV group, and 257 in the S-1 group). The full-cohort data consisted of 463 patients with metastatic PC, whereas the complete-case dataset consisted of 446 patients. Unadjusted patients’ baseline characteristics are summarized in Table 1. S-1 tended to be given to patients with poor performance status (PS) and moderate or more ascites. In contrast, nal-IRI + 5-FU/LV tended to be given to patients with younger age and liver metastasis. The propensity score distribution between the treatment groups showed covariate imbalance before IPTW adjustment (Supplementary Fig. 1). Standardized mean differences of unadjusted comparisons showed potential imbalances between both treatment groups with respect to many clinical characteristics of interest (e.g., age and Eastern Cooperative Oncology Group PS) (Table 1, Supplemental Fig. 2).

**Table 1:** Patients’ baseline characteristics

		nal-IRI + 5-FU/LV	S-1	p value		Standardized mean difference		Variance ratio	
		(n = 206)	(n = 257)			Before adjustment	After adjustment	Before adjustment	After adjustment
Propensity score									
						1.021	0.0543	0.681	0.739
Age group, y				< 0.001					
	< 50	6 (2.9)	7 (2.7)			0.002	− 0.002		
	50—59	37 (18.0)	16 (6.2)			0.117	0.002		
	60—69	57 (27.7)	65 (25.3)			0.024	< 0.001		
	70—79	100 (48.5)	131 (51.0)			− 0.024	0.009		
	≥ 80	6 (2.9)	38 (14.8)			− 0.119	− 0.009		
Sex				0.042					
	Male (%)	104 (50.5)	154 (59.9)						
	Female (%)	102 (49.5)	103 (40.1)			0.094	0.003		
ECOG performance status				0.005					
	0	91 (44.2)	78 (30.4)			0.135	0.028		
	1	101 (49.0)	142 (55.3)			− 0.074	− 0.016		
	≥ 2	13 (6.3)	31 (12.1)			− 0.061	− 0.013		
	Missing	1 (0.5)	6 (2.3)						
Hospital type				< 0.001					
	Community hospital	21 (10.2)	60 (23.4)			− 0.132	− 0.003		
	Cancer center	105 (51.0)	131 (51.0)			− 0.000	− 0.008		
	University hospital	80 (38.8)	66 (25.7)			0.132	0.011		
Ascites				0.021					
	None	144 (69.9)	156 (60.7)			0.097	0.010		
	Mild	47 (22.8)	64 (24.9)			− 0.021	0.001		
	Moderate or more	14 (6.8)	37 (14.4)			− 0.076	− 0.011		
	Missing	1 (0.5)	0						
Biliary drainage				0.097					
	Yes	55 (26.7)	87 (33.9)			− 0.072	− 0.011		
	No	151 (73.3)	170 (66.2)						
Disease status				0.005					
	Metastatic	174 (84.5)	238 (92.6)						
	Recurrent	32 (15.5)	19 (7.4)			0.081	0.002		
Previous therapy				< 0.001					
	Gemcitabine	2 (1.0)	30 (11.7)			− 0.107	− 0.013		
	Gemcitabine plus nab-paclitaxel	203 (98.5)	227 (88.3)			0.102	0.011		
	Investigational therapy	1 (0.5)	0			0.005	0.002		
Tumor location				0.865					
	Head	93 (45.2)	114 (44.4)						
	Other^‡^	113 (54.9)	143 (55.6)			− 0.008	0.019		
Liver metastasis				0.019					
	Yes	136 (66.0)	142 (55.3)			0.108	0.030		
	No	70 (34.0)	115 (44.8)						
Total bilirubin, mg/dL				0.688					
	≤ 1.5	203 (98.5)	252 (98.1)						
	> 1.5	3 (1.5)	5 (2.0)			− 0.005	− 0.005		
Albumin, g/dL				0.115					
	< 3.5	81 (39.3)	118 (45.9)			− 0.075	0.002		
	≥ 3.5	125 (60.7)	135 (52.5)						
	Missing	0	4 (1.6)						
C-reactive protein, mg/dL				0.911					
	≤ 1.0	132 (64.1)	165 (64.2)						
	> 1.0	72 (35.0)	92 (35.8)			− 0.007	− 0.017		
	Missing	2 (1.0)	0						
Hemoglobin, g/dL				0.854					
	< 10.0	69 (33.5)	84 (32.7)			0.008	− 0.021		
	≥ 10.0	137 (66.5)	173 (67.3)						
Carbohydrate antigen 19–9, U/mL				0.422					
	< 1,000	105 (51.0)	139 (54.1)						
	≥ 1,000	101 (49.0)	115 (44.8)			0.057	− 0.004		
	Missing	0	3 (1.2)						
Creatinine clearance, mL/min				0.261					
	< 50	26 (12.6)	42 (16.3)			− 0.037	0.037		
	≥ 50	180 (87.4)	215 (83.7)						
Body mass index, kg/m^2^				0.335					
	< 18.5	121 (58.7)	168 (65.4)			− 0.066	− 0.011		
	18.5—24.9	50 (24.3)	51 (19.8)			0.044	0.009		
	≥ 25.0	35 (17.0)	38 (14.8)			0.022	0.002		
UGT1A1 *6/*28 status									
	Polymorphism	31 (15.0)							
	UGT1A1 *6/*6	11 (5.3)							
	UGT1A1 *28/*28	11 (5.3)							
	UGT1A1 *6/*28	10 (4.9)							

^†^Values were computed in non-missing patients

^‡^Body, tail, or unknown location

An absolute value of the standardized mean difference less than 0.1 and that of the variance ratio less than 2 were considered to be well-balanced

**Fig. 2:**
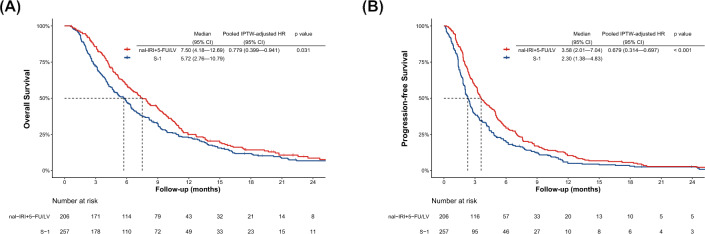
Kaplan–Meier curves comparing overall survival **A**, and progression-free survival **B** in metastatic pancreatic cancer patients treated with nal-IRI + 5-FU/LV or S-1 monotherapy in the full-cohort dataset nal-IRI + 5-FU/LV, liposomal irinotecan plus fluorouracil/folinic acid; *HR* hazard ratio, *IPTW* inverse probability of treatment weighting

### Treatment efficacy in the full-cohort data

At the clinical data cutoff (September 25, 2023), the median follow-up period was 5.85 months (interquartile range 2.60–11.31 months). During the follow-up period, 173 patients in the nal-IRI + 5-FU/LV group and 224 patients in the S-1 group died. Kaplan–Meier curves showed that median OS was significantly longer for the nal-IRI + 5-FU/LV group (7.50 months; 95% CI 4.18—12.69 months) than for the S-1 group (5.72 months; 95% CI 2.76—10.79 months) (*p* = 0.031) (Fig. 2A). The 1-year OS rates were 25.0% for the nal-IRI + 5-FU/LV group and 22.7% for the S-1 group.

Tumor responses in the nal-IRI + 5-FU/LV and S-1 groups are shown in Table 2. A complete or partial response was observed in 16 of the 206 patients in the nal-IRI + 5-FU/LV group and 15 of the 257 patients in the S-1 group. Although there was no significant difference in the objective response rate between the nal-IRI + 5-FU/LV group and the S-1 group (7.8% vs. 5.8%, *p* = 0.409), the disease control rate was significantly higher in the nal-IRI + 5-FU/LV group than in the S-1 group (55.3% vs. 35.8%, *p* < 0.001).

**Table 2:** Tumor response by regimen

		nal-IRI + 5-FU/LV	S-1	p value
		(n = 206)	(n = 257)	
**Best overall response**				
	Complete response (%)	0	1 (0.4)	
	Partial response (%)	16 (7.8)	14 (5.5)	
	Stable disease (%)	98 (47.6)	77 (30.0)	
	Progressive disease (%)	84 (40.8)	140 (54.5)	
	Not evaluable (%)	8 (3.9)	25 (9.7)	
**Objective response rate (%)**		7.8	5.8	0.409
**(95% CI)**		(4.5—12.3)	(3.3—9.4)	
**Disease control rate (%)**		55.3	35.8	< 0.001
**(95% CI)**		(48.3—62.3)	(29.9—42.0)	

CI, confidence interval

During follow-up, 193 patients in the nal-IRI + 5-FU/LV group and 238 patients in the S-1 group progressed or died. Kaplan–Meier curves showed that median PFS was significantly longer for the nal-IRI + 5-FU/LV group (3.58 months; 95% CI 2.01—7.04 months) than for the S-1 group (2.30 months; 95% CI 1.38—4.83 months) (*p* < 0.001) (Fig. 2B). The 1-year PFS rates were 10.3% for the nal-IRI + 5-FU/LV group and 4.9% for the S-1 group.

### Treatment efficacy based on IPTW analysis with multiple imputation

In the IPTW-adjusted Cox proportional hazards model for multiple imputed datasets, nal-IRI + 5-FU/LV was associated with a significant OS benefit (pooled IPTW-adjusted HR, 0.779; 95% CI 0.399–0.941; *p* = 0.025). The propensity score distribution between the treatment groups achieved adequate balance after IPTW adjustment (Supplemental Fig. 1). After IPTW adjustment, all absolute standardized mean differences were less than 0.1, which indicated that patients who received nal-IRI + 5-FU/LV or S-1 were balanced (Table 1**, **Supplemental Fig. 2). The variance ratio of the propensity score between the treatment groups showed improved covariate balance after IPTW adjustment (Table 1), and nal-IRI + 5-FU/LV was also associated with a significant PFS benefit (IPTW-adjusted HR 0.679; 95% CI 0.314—0.697; *p* < 0.001) in the IPTW-adjusted Cox proportional hazards model for multiple imputation datasets.

### Treatment efficacy in the complete-case analysis dataset

**Fig. 3:**
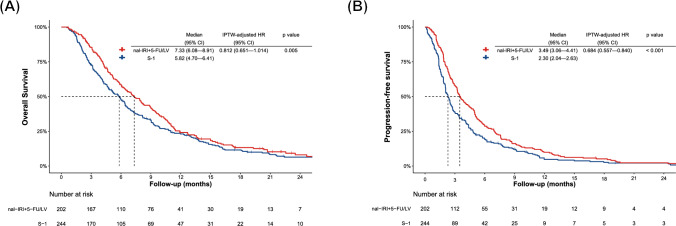
Kaplan–Meier curves comparing overall survival **A**, and progression-free survival **B** in metastatic pancreatic cancer patients treated with nal-IRI + 5-FU/LV or S-1 monotherapy in the complete-case analysis dataset.nal-IRI + 5-FU/LV, liposomal irinotecan plus fluorouracil/folinic acid, *HR* hazard ratio, *IPTW* inverse probability of treatment weighting

In the complete-case dataset that consisted of 446 patients, Kaplan–Meier curves showed that median OS was significantly longer for the nal-IRI + 5-FU/LV group (7.33 months; 95% CI 6.08–8.91 months) than for the S-1 group (5.82 months; 95% CI 4.70–6.41 months) (*p* = 0.005) (Fig. 3A). In the IPTW-adjusted Cox proportional hazards model for the complete-case dataset, nal-IRI + 5-FU/LV was associated with a borderline significant OS benefit (HR 0.812; 95% CI 0.651–10.14; *p* = 0.067).

Kaplan–Meier curves also showed that median PFS was significantly longer for the nal-IRI + 5-FU/LV group (3.49 months; 95% CI 3.06–4.41 months) than for the S-1 group (2.30 months; 95% CI 2.04–2.63 months) (*p* < 0.001) (Fig. 3B). In the IPTW-adjusted Cox proportional hazards model for the complete-case dataset, nal-IRI + 5-FU/LV was associated with a significant PFS benefit (HR 0.679; 95% CI 0.314–0.697; *p* < 0.001).

### Subsequent treatment

Overall, 94 (45.6%) of 206 patients in the nal-IRI + 5-FU/LV group and 42 (16.3%) of 257 patients in the S-1 group received subsequent treatment (Supplemental Table 1). The most common subsequent treatments were FOLFOX (51 patients [24.8%]) in the nal-IRI + 5-FU/LV group and nal-IRI + 5-FU/LV (21 patients [8.2%]) in the S-1 group.

### Exploratory subgroup analysis

**Fig. 4:**
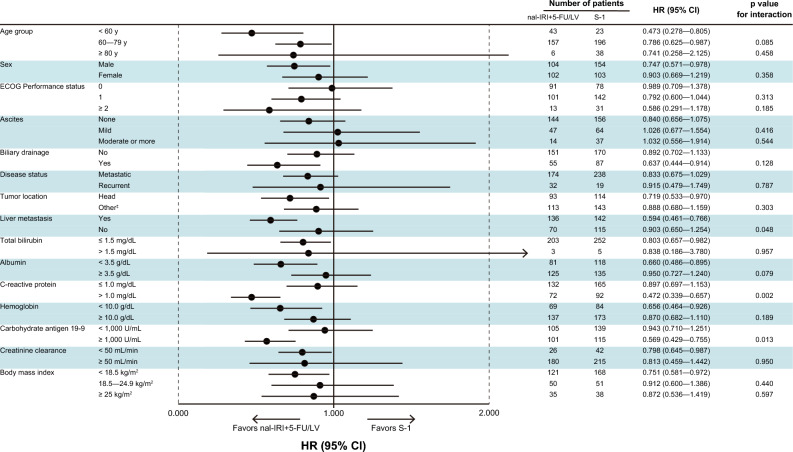
Forest plot of the treatment effect on overall survival by baseline factors nal-IRI + 5-FU/LV, liposomal irinotecan plus fluorouracil/folinic acid; *HR* hazard ratio, *CI* confidence interval

The results of subgroup analyses of OS according to baseline factors are shown in Fig. 4. In the younger age subgroups (< 60 years and 60—79 years) and the liver metastasis subgroup, nal-IRI + 5-FU/LV showed favorable HR for OS compared to S-1. The HRs for OS of nal-IRI + 5-FU/LV to S-1 were as follows: 0.473 (95% CI 0.278–0.805) in the < 60-year subgroup and 0.786 (95% CI 0.625–0.987) in the 60–79-year subgroup; and 0.594 (95% CI 0.461–0.766) in the liver metastasis subgroup.

## Discussion

In the present study using multicenter, retrospective data, OS was compared using the IPTW method between patients treated with nal-IRI + 5-FU/LV and those treated with S-1 monotherapy. The median OS was significantly longer for the nal-IRI + 5-FU/LV group than for the S-1 group. In the IPTW-adjusted Cox proportional hazards model for the multiple imputed dataset, nal-IRI + 5-FU/LV was associated with a significant OS benefit. The median PFS was also significantly longer in the nal-IRI + 5-FU/LV group than in the S-1 group; however, the response rate was not significantly different between the groups. Although the nal-IRI + 5-FU/LV regimen was approved in Japan based on the NAPOLI-1 study [11] and a randomized phase 2 trial [18] comparing it to 5-FU/LV, a direct comparison with the previous Japanese community standard treatment, S-1, has not been conducted. The present study showed that nal-IRI + 5-FU/LV can offer longer survival for PC patients after gemcitabine-based chemotherapy than S-1 monotherapy as a second-line treatment.

Determining the optimal second-line treatment remains challenging in the daily management of medically fit patients with metastatic PC. This is primarily due to the lack of comparison data of chemotherapy regimens. Thus, the choice of second-line treatment was determined empirically based on first-line treatment. At present, nal-IRI + 5-FU/LV is the only clear evidence-based, second-line treatment for metastatic PC; the nal-IRI + 5-FU/LV regimen significantly improved OS compared with the 5-FU/LV regimen in the NAPOLI-1 study [11]. However, several fluorouracil-based treatment regimens, other than 5-FU/LV regimen, have been used as second-line treatments for PC. The ESMO guideline still recommends oxaliplatin in combination with the 5-FU/LV regimen as an alternative option to the nal-IRI + 5-FU/LV for PC after gemcitabine-based first-line treatment [13], since randomized trials of oxaliplatin in combination with the 5-FU/LV regimen have generated conflicting data [9] [10] [15]. Theoretically, the FOLFIRINOX regimen may have a stronger antitumor effect even in second-line treatment. However, a multicenter, retrospective study by Park et al. reported that second-line FOLFIRINOX did not provide any survival benefit over the nal-IRI + 5-FU/LV regimen [32]. In Asia, S-1 has been widely used historically as a second-line treatment for metastatic PC after gemcitabine-based treatment [33]. One of its advantages is that it does not require continuous infusion or intravenous port implantation, contributing to its common use in clinical practice. However, the present study showed that the nal-IRI + 5-FU/LV regimen can offer longer survival for PC patients after gemcitabine-based treatment than S-1 monotherapy. In 2023, the JCOG1611 study comparing gemcitabine plus nab-paclitaxel, modified FOLFIRINOX, and the S-IROX regimen in metastatic/recurrent PC was reported, wherein modified FOLFIRINOX failed to show superiority over gemcitabine plus nab-paclitaxel in terms of OS [8]. Furthermore, grade ≥ 3 gastrointestinal toxicities were more frequent in the modified FOLFIRINOX arms than in the gemcitabine plus nab-paclitaxel arm, suggesting that even more patients will be treated with gemcitabine plus nab-paclitaxel rather than FOLFIRINOX. The present study will contribute to optimizing the therapeutic approach after gemcitabine plus nab-paclitaxel.

The present results also indicated trends in second-line treatment selection in Japan. Importantly, nal-IRI + 5-FU/LV was frequently used for patients with younger age or liver metastasis. These trends seem reasonable, given that nal-IRI + 5-FU/LV showed a good HR compared with S-1 in the subsets of patients with younger age or liver metastasis. Conversely, S-1 was frequently used for patients with ascites. The poor prognosis and limited efficacy of chemotherapy in patients with ascites have been well documented [34] [35]. In fact, nal-IRI + 5-FU/LV showed a modest HR compared with S-1 in the subset of patients with ascites in the present exploratory subgroup analysis. Based on these findings, the benefit of nal-IRI + 5-FU/LV in patients with ascites is considered relatively limited. Furthermore, ascites can impair the quality of life of PC patients [36]. One potential disadvantage of nal-IRI + 5-FU/LV is that this regimen requires a central venous port and continuous infusion for 46 h, which may cause psychological stress for patients [37]. In addition, Moss reported that a central venous port had fewer safety and cost advantages in patients receiving chemotherapy for ≤ 3 months [38]. In contrast, S-1 is administered orally. Therefore, for patients with a poor prognosis, such as those with ascites, S-1 may be preferable to nal-IRI + 5-FU/LV due to its simple administration and lack of requirement for a central venous port.

The present study had some limitations. The first is a lack of safety and dose modification data. These data were not collected because safety data are unreliable in such retrospective studies because of their retrospective nature. Therefore, whether nal-IRI + 5-FU/LV or S-1 is beneficial based on a direct comparison of safety data could not be evaluated. However, safety data for both nal-IRI + 5-FU/LV and S-1 monotherapy are well known based on many prospective trials [11] [15] [16] [17] [18] [33]. These data support clinician’s decision-making in second-line treatment selection for PC based on safety considerations. The second limitation is that the present analysis was a retrospective study that lacked adequate statistical power and was furthermore subject to various forms of bias. To address the limitations of the retrospective study design, IPTW analysis and multiple imputation were used to minimize the impact of selection bias and missing data. In this study, a balance check (standardized mean difference and the variance ratio) demonstrated that patients’ baseline characteristics were well-balanced between the nal-IRI + 5-FU/LV and S-1 groups. These findings ensure the quality of the matching process and a well-balanced comparison of patients’ baseline characteristics between the two groups. On the other hand, nal-IRI + 5-FU/LV showed a favorable trend in HR for OS compared with S-1 in the complete-case analysis dataset, but a statistical significance showed marginal in contrast to multiple imputed dataset. This fact suggests that the robustness of this study still needs to be addressed. The third limitation is that we did not collect detailed data on perioperative treatment, especially for fluorouracil-based regimens. We excluded recurrence patients refractory to adjuvant chemotherapy, but prior use of fluorouracil-based regimen in neoadjuvant or adjuvant setting may possibly affect the anticancer effect of nal-IRI + 5-FU/LV and S-1. The fourth limitation is the timing of this study. While nal-IRI + 5-FU/LV is a relatively new regimen, S-1 has been widely used historically. Thus, we defined the eligible study period as just before and after the approval of nal-IRI + 5-FU/LV to minimize differences in historical background. However, these differences could not be entirely avoided. In addition, the possibility remains that the COVID-19 pandemic may have influenced treatment decision-making and outcomes in patients. The final limitation is that the efficacy of nal-IRI + 5-FU/LV in small subpopulations, such as elderly patients ≥ 80 years old, remains unclear. It is common practice to exclude these small subpopulations from clinical trials, which often results in a lack of reliable data. IPTW analysis is an efficient approach for balancing overall patients’ baseline characteristics and for comparing treatment effects between two groups. However, this approach is not suitable for assessing treatment effects in small subpopulations. Consequently, subgroup analyses were performed, but due to small sample sizes, the treatment effects of nal-IRI + 5-FU/LV for such small subpopulations remain unclear.

## Conclusion

The present study supports the use of nal-IRI + 5-FU/LV as a standard second-line treatment for PC patients after gemcitabine-based chemotherapy.

## Supplementary Information

Below is the link to the electronic supplementary material.

Supplementary file1 (PDF 64 KB)

Supplementary file2 (EPS 1530 KB)

Supplementary file3 (EPS 1433 KB)

Supplementary file4 (DOCX 30 KB)
