# Correction: Efficacy of liposomal irinotecan + 5-FU/LV vs. S-1 in gemcitabine-refractory metastatic pancreatic cancer: a real-world study using inverse probability of treatment weighting

**DOI:** 10.1007/s00535-025-02215-1

**Published:** 2025-01-29

**Authors:** Hiroshi Imaoka, Masafumi Ikeda, Satoshi Kobayashi, Akihiro Ohba, Masayuki Ueno, Yuko Suzuki, Hidetaka Tsumura, Nana Kimura, Shinya Kawaguchi, Yasuyuki Kawamoto, Kohei Nakachi, Kunihiro Tsuji, Noritoshi Kobayashi, Reiko Ashida, Naohiro Okano, Kumiko Umemoto, Gou Murohisa, Ayumu Hosokawa, Akinori Asagi, Hiroko Nebiki, Rei Suzuki, Takeshi Terashima, Ryusuke Shibata, Kazuhito Kawata, Toshifumi Doi, Hiroshi Ohyama, Yohei Kitano, Kazuhiko Shioji, Hiroyuki Okuyama, Atsushi Naganuma, Yuji Negoro, Yasunari Sakamoto, Satoshi Shimizu, Chigusa Morizane, Makoto Ueno, Junji Furuse, Hiroaki Nagano, the Japan Oncology Network in Hepatobiliary and Pancreas

**Affiliations:** 1 Department of Hepatobiliary and Pancreatic Oncology, National Cancer Center Hospital East, Kashiwa, Japan; 2 Department of Gastroenterology, Kanagawa Cancer Center, Yokohama, Japan; 3 Department of Hepatobiliary and Pancreatic Oncology, National Cancer Center Hospital, Tokyo, Japan; 4 Division of Gastrointestinal Oncology, Shizuoka Cancer Center, Shizuoka, Japan; 5 Department of Gastroenterology and Hepatology, Kurashiki Central Hospital, Kurashiki, Japan; 6 Department of Gastroenterology and Hepatology, Graduate School of Medicine, Kyoto University, Kyoto, Japan; 7 Department of Gastroenterology, Saitama Cancer Center, Saitama, Japan; 8 Department of Gastroenterological Oncology, Hyogo Cancer Center, Akashi, Japan; 9 Department of Surgery and Science, Faculty of Medicine, University of Toyama, Toyama, Japan; 10 Department of Gastroenterology, Shizuoka General Hospital, Shizuoka, Japan; 11 Division of Cancer Center, Hokkaido University Hospital, Sapporo, Japan; 12 Department of Medical Oncology, Tochigi Cancer Center, Utsunomiya, Japan; 13 Department of Gastroenterology, Ishikawa Prefectural Central Hospital, Kanazawa, Japan; 14 Gastroenterological Center, Yokohama City University Medical Center, Yokohama, Japan; 15 Second Department of Internal Medicine, Wakayama Medical University, Wakayama, Japan; 16 Department of Medical Oncology, Kyorin University Faculty of Medicine, Tokyo, Japan; 17 Department of Clinical Oncology, St. Marianna University School of Medicine, Kawasaki, Japan; 18 Department of Gastroenterology, Seirei Hamamatsu General Hospital, Hamamatsu, Japan; 19 Department of Clinical Oncology, University of Miyazaki Hospital, Miyazaki, Japan; 20 Department of Gastrointestinal Medical Oncology, National Hospital Organization Shikoku Cancer Center, Matsuyama, Japan; 21 Department of Gastroenterology, Osaka City General Hospital, Osaka, Japan; 22 Department of Gastroenterology, Fukushima Medical University, Fukushima, Japan; 23 Department of Gastroenterology, Kanazawa University Hospital, Kanazawa, Japan; 24 Digestive and Lifestyle Diseases, Kagoshima University Graduate School of Medical and Dental Sciences, Kagoshima, Japan; 25 Hepatology Division, Department of Internal Medicine II, Hamamatsu University School of Medicine, Hamamatsu, Japan; 26 Department of Molecular Gastroenterology and Hepatology, Kyoto Prefectural University of Medicine, Kyoto, Japan; 27 Department of Gastroenterology, Chiba University, Chiba, Japan; 28 Division of Gastroenterology, Department of Medicine, Asahikawa Medical University, Asahikawa, Japan; 29 Department of Internal Medicine, Niigata Cancer Center Hospital, Niigata, Japan; 30 Department of Medical Oncology, Kagawa University Hospital, Miki, Japan; 31 Department of Gastroenterology, National Hospital Organization Takasaki General Medical Center, Gunma, Japan; 32 Department of Oncological Medicine, Kochi Health Sciences Center, Kochi, Japan; 33 Department of Gastroenterology and Hepatology, International University of Health and Welfare Atami Hospital, Shizuoka, Japan; 34 Department of Gastroenterological, Breast and Endocrine Surgery, Yamaguchi University Graduate School of Medicine, Ube, Japan

**Correction: Journal of Gastroenterology** 10.1007/s00535-024-02186-9

The article ‘Efficacy of liposomal irinotecan + 5‑FU/LV vs. S‑1 in gemcitabine‑refractory metastatic pancreatic cancer: a real‑world study using inverse probability of treatment weighting’, written by ‘Hiroshi Imaoka, Masafumi Ikeda, Satoshi Kobayashi, Akihiro Ohba, Masayuki Ueno, Yuko Suzuki, Hidetaka Tsumura, Nana Kimura, Shinya Kawaguchi, Yasuyuki Kawamoto, Kohei Nakachi, Kunihiro Tsuji, Noritoshi Kobayashi, Reiko Ashida, Naohiro Okano, Kumiko Umemoto, Gou Murohisa, Ayumu Hosokawa, Akinori Asagi, Hiroko Nebiki, Rei Suzuki, Takeshi Terashima, Ryusuke Shibata, Kazuhito Kawata, Toshifumi Doi, Hiroshi Ohyama, Yohei Kitano, Kazuhiko Shioji, Hiroyuki Okuyama, Atsushi Naganuma, Yuji Negoro, Yasunari Sakamoto, Satoshi Shimizu, Chigusa Morizane, Makoto Ueno, Junji Furuse, Hiroaki Nagano, the Japan Oncology Network in Hepatobiliary and Pancreas’, was originally published under exclusive license to Japanese Society of Gastroenterology. As a result of the subsequent decision to publish the article under the open access model, the article’s copyright notice was changed on 27 December 2024 to © The Author(s) 2024 and the article is now distributed under a Creative Commons Attribution 4.0 International License, which permits use, sharing, adaptation, distribution and reproduction in any medium or format, as long as you give appropriate credit to the original author(s) and the source, provide a link to the Creative Commons licence, and indicate if changes were made. The images or other third party material in this article are included in the article’s Creative Commons licence, unless indicated otherwise in a credit line to the material. If material is not included in the article’s Creative Commons licence and your intended use is not permitted by statutory regulation or exceeds the permitted use, you will need to obtain permission directly from the copyright holder. To view a copy of this licence, visit http://creativecommons.org/licenses/by/4.0/.

The original article has been corrected.

