# Supplementary material for: Efficacy of liposomal irinotecan + 5-FU/LV vs. S-1 in gemcitabine-refractory metastatic pancreatic cancer: a real-world study using inverse probability of treatment weighting
Source: J Gastroenterol. 2024 Nov 30;60(3):356–67. doi: 10.1007/s00535-024-02186-9 (PMC11880175; doi:10.1007/s00535-024-02186-9)
Supplement: Supplementary file 1 — Supplementary file1 (PDF 64 KB) [file 535_2024_2186_MOESM1_ESM.pdf]

Supplemental Appendix

Statistical methods

All patients who met the inclusion criteria at baseline were included as the full-cohort data, and their baseline characteristics in the nal-IRI+5-FU/LV and S-1 groups were categorized and compared using the chi-squared test. The time-to-event distribution was estimated using the Kaplan-Meier method, and the log-rank test was used to evaluate the differences between the nal-IRI+5-FU/LV and S-1 groups in the full-cohort data.

This study has two potential biases due to its retrospective design. One is missing data, and the other is selection bias. Due to the retrospective design, covariate data were often missing. Missing data not only reduce the sample size, but may also lead to under- or over-estimation of treatment effects due to incomplete data. For example, patients with advanced disease may need to initiate second-line treatment without sufficient assessment, such as carbohydrate antigen 19-9 (CA19-9) and albumin. Selection bias, mainly caused by physician or patient choice, can affect the outcome of chemotherapy. For example, patients with poor PS tend to avoid combination chemotherapy such as

nal-IRI+5-FU/LV [12]. To minimize these biases in this study, inverse probability of treatment weighting (IPTW) analysis with multiple imputation was performed.

When dealing with missing data, multiple imputation was performed under the assumption that the data were missing at random. Specifically, Rubin's rules [22] were used to generate pooled effect estimates and variances across imputed datasets [23]. For multiple imputation, the logistic regression method was used for binary categorical covariates, and polytomous logistic regression methods were used for nominal categorical covariates. Five multiple imputation iterations were conducted, consistent with both the most commonly used guideline [22] and the most recent guidelines, which recommend conducting the same number of imputations as the average percentage rate of missingness for the data [24] [25]. In this study, the average percentage of missing data for 17 baseline factors was 0.2%. The highest percentages of missing data were 1.5% for ECOG performance status (PS) and 0.9% for albumin. Therefore, five iterations were considered satisfactory for this study. Following multiple imputation, a complete-case analysis was also performed for survival outcomes using only cases with complete data, with no imputation, as a sensitivity analysis

to validate the imputation model and evaluate the reliability of the results in accordance with the guideline [26].

In IPTW analysis, the weights are calculated as the inverse of the propensity score between the patient groups treated with nal-IRI+5-FU/LV and with S-1. The covariates included in the IPTW analysis were age (< 50, 50—59, 60—69, 70—79, or ≥ 80 years), sex (male or female), ECOG PS (0, 1, or ≥ 2), hospital type (community hospital, cancer center, or university hospital), ascites (none, mild, or moderate or more), biliary drainage (yes or no), disease status (metastatic or recurrent), previous therapy (gemcitabine, nab-paclitaxel plus gemcitabine, or investigational therapy), tumor location (head or other), liver metastasis (yes or no), total bilirubin (≤ 1.5 mg / dL or > 1.5 mg / dL), albumin (< 3.5 g/dL or ≥ 3.5 g/dL), C-reactive protein (≤ 1.0 mg/dL or > 1.0 mg/dL), hemoglobin (< 10.0 g/dL or ≥ 10.0 g/dL), CA19-9 (< 1,000 U/mL or ≥ 1,000 U/mL), creatinine clearance (< 50 mL/min or ≥ 50 mL/min), and body mass index (< 18.5, 18.5—24.9, or ≥ 25.0 kg/m^2). To evaluate the balance of covariates between the nal-IRI+5-FU/LV and S-1 groups, standardized mean differences and variance ratios were used. An absolute value of the standardized mean difference less than 0.1 and that of the variance ratio less than 2 were considered to be well

balanced. A significance test was not used for assessment of balance, because the p value depends on the sample size; the sample size of the study population after weighting is reduced, and thus the power to detect significant differences is also decreased [27] [28].

In multiple imputation datasets, imputed IPTW-adjusted hazard ratios (HRs) and corresponding 95% confidence intervals (CIs) were calculated using a Cox proportional hazards model and combined into pooled estimates. In complete-case analysis datasets, IPTW-adjusted HRs and corresponding 95% CIs were calculated using a Cox proportional hazards model, and p values were calculated using an IPTW-adjusted log-rank test.

Exploratory subgroup analysis was performed to evaluate the interactions between baseline factors and treatment effect on OS; HRs for the nal-IRI+5-FU/LV group vs. the S-1 group with their 95% CIs were estimated using a Cox proportional hazards model for each subgroup without missing values.

Data were analyzed using STATA version 15.1 (StataCorp, College Station, TX, USA) and R version 4.3.2. (http://www.r-project.org/). The IPTW analysis and IPTW analysis with multiple imputations were performed using the WeightIt package version 0.14.2 [29] and MatchThem package version 1.1 in R

[30], respectively. Covariate balance was assessed using Cobalt package version 4.5.1 in R [31]. All reported p values are two-sided.

R codes for estimation of hazard ratios for overall survival and progression-free survival by inverse probability of treatment weighting analysis with multiple imputation

// Estimation of hazard ratios for overall survival by inverse probability of treatment weighting analysis with multiple imputation

```
DATA <-
read.csv('DATA_OS3.csv',header=T,stringsAsFactor=F,fileEncoding='UTF-8-
BOM')
DATA$Sex<- factor(DATA$Sex, labels=c('Male','Female'))
DATA$Age_strata<-
factor(DATA$Age_strata,labels=c('Age<50','50≤Age<60','60≤Age<70','70≤Age<
80','80≤Age'))
DATA$Prior_treatment<-
factor(DATA$Prior_treatment,labels=c('Gem','Gem+nab_PTX','Investigational_tr
eatment'))
DATA$Ascites<- factor(DATA$Ascites, labels=c('None','Mild','Moderate or
more'))
DATA$Biliary_drainage<- factor(DATA$Biliary_drainage, labels=c('No','Yes'))
```

```
DATA$Prior_surgical_resection<-
factor(DATA$Prior_surgical_resection,labels=c('No','Yes'))
DATA$Liver_met<- factor(DATA$Liver_met, labels=c('No','Yes'))
DATA$Hospital_type<- factor(DATA$Hospital_type, labels=c('Community
hospital','Cancer center','University hospital'))
DATA$Tumor_location2<-
factor(DATA$Tumor_location2,labels=c('Head','Body_tail_or_unknown'))
DATA$ECOG_PS2<- factor(DATA$ECOG_PS2,labels=c('PS_0','PS_1','PS≥2'))
DATA$T_bil_strata<- factor(DATA$T_bil_strata,labels=c('T_bil≤1.5','T_bil>1.5'))
DATA$Alb_strata<- factor(DATA$Alb_strata,labels=c('Alb≥3.5','Alb<3.5'))
DATA$CRP_strata<- factor(DATA$CRP_strata,labels=c('CRP≤1.0','CRP>1.0'))
DATA$Hb_strata<- factor(DATA$Hb_strata,labels=c('Hb≥10','Hb<10'))
DATA$CA19_9_strata<-
factor(DATA$CA19_9_strata,labels=c('CA19_9<1000','CEA≥1000'))
DATA$CCr_strata<- factor(DATA$CCr_strata,labels=c('CCr≥50','CCr<50'))
DATA$BMI_strata<-
factor(DATA$BMI_strata,labels=c('BMI≤18.5','BMI_Normal','BMI≥25.0'))
DATA$Treatment<- factor(DATA$Treatment, labels=c('S-1','nal-IRI+5-FU/LV'))
```

```
DATA$Best_response<-
factor(DATA$Best_response,labels=c('CR','PR','SD','PD','NE'))

MI_data<-
mice(DATA,m=5,maxit=20,method=list(Sex='logreg',Prior_treatment='polyreg',A
scites='polyreg',Biliary_drainage='logreg',Prior_surgical_resection='logreg',Liver
_met='logreg',Hospital_type='polyreg',Tumor_location2='logreg',Age_strata='pol
yreg',ECOG_PS2='polyreg',T_bil_strata='logreg',Alb_strata='logreg',CRP_strata
='logreg',Hb_strata='logreg',CA19_9_strata='logreg',CCr_strata='logreg',BMI_str
ata='polyreg',Treatment='logreg',Best_response='polyreg',Event_OS='polyreg',T
ime_OS='pmm',Event_PFS='',Time_PFS=''),seed=12345)

iptw1<-
weightthem(Treatment~Sex+Prior_treatment+Ascites+Biliary_drainage+Prior_s
urgical_resection+Liver_met+Hospital_type+Tumor_location2+Age_strata+ECO
G_PS2+T_bil_strata+Alb_strata+CRP_strata+Hb_strata+CA19_9_strata+CCr_
strata+BMI_strata,MI_data,approach='within',method='ps')
```

```
result_iptw <-
with(iptw1,coxph(Surv(Time_OS,Event_OS)~Treatment),cluster=TRUE)
result_iptw
pool_result<- pool(result_iptw)
summary(pool_result,conf.int=TRUE)
```

// Estimation of hazard ratios for progression-free survival by inverse probability of treatment weighting analysis with multiple imputation

```
DATA <-
read.csv('DATA_PFS.csv',header=T,stringsAsFactor=F,fileEncoding='UTF-8-
BOM')
DATA$Sex<- factor(DATA$Sex, labels=c('Male','Female'))
DATA$Age_strata<-
factor(DATA$Age_strata,labels=c('Age<50','50≤Age<60','60≤Age<70','70≤Age<
80','80≤Age'))
DATA$Prior_treatment<-
factor(DATA$Prior_treatment,labels=c('Gem','Gem+nab_PTX','Investigational_tr
eatment'))
```

```
DATA$Ascites<- factor(DATA$Ascites, labels=c('None','Mild','Moderate or
more'))
DATA$Biliary_drainage<- factor(DATA$Biliary_drainage, labels=c('No','Yes'))
DATA$Prior_surgical_resection<-
factor(DATA$Prior_surgical_resection,labels=c('No','Yes'))
DATA$Liver_met<- factor(DATA$Liver_met, labels=c('No','Yes'))
DATA$Hospital_type<- factor(DATA$Hospital_type, labels=c('Community
hospital','Cancer center','University hospital'))
DATA$Tumor_location2<-
factor(DATA$Tumor_location2,labels=c('Head','Body_tail_or_unknown'))
DATA$ECOG_PS2<- factor(DATA$ECOG_PS2,labels=c('PS_0','PS_1','PS≥2'))
DATA$T_bil_strata<- factor(DATA$T_bil_strata,labels=c('T_bil≤1.5','T_bil>1.5'))
DATA$Alb_strata<- factor(DATA$Alb_strata,labels=c('Alb≥3.5','Alb<3.5'))
DATA$CRP_strata<- factor(DATA$CRP_strata,labels=c('CRP≤1.0','CRP>1.0'))
DATA$Hb_strata<- factor(DATA$Hb_strata,labels=c('Hb≥10','Hb<10'))
DATA$CA19_9_strata<-
factor(DATA$CA19_9_strata,labels=c('CA19_9<1000','CEA≥1000'))
DATA$CCr_strata<- factor(DATA$CCr_strata,labels=c('CCr≥50','CCr<50'))
```

```
DATA$BMI_strata<-
factor(DATA$BMI_strata,labels=c('BMI≤18.5','BMI_Normal','BMI≥25.0'))
DATA$Treatment<- factor(DATA$Treatment, labels=c('S-1','nal-IRI+5-FU/LV'))
DATA$Best_response<-
factor(DATA$Best_response,labels=c('CR','PR','SD','PD','NE'))

MI_data<-
mice(DATA,m=5,maxit=20,method=list(Sex='logreg',Prior_treatment='polyreg',A
scites='polyreg',Biliary_drainage='logreg',Prior_surgical_resection='logreg',Liver
_met='logreg',Hospital_type='polyreg',Tumor_location2='logreg',Age_strata='pol
yreg',ECOG_PS2='polyreg',T_bil_strata='logreg',Alb_strata='logreg',CRP_strata
='logreg',Hb_strata='logreg',CA19_9_strata='logreg',CCr_strata='logreg',BMI_str
ata='polyreg',Treatment='logreg',Best_response='polyreg',Event_PFS='polyreg',
Time_PFS='pmm'),seed=123456)

iptw1<-
weightthem(Treatment~Sex+Prior_treatment+Ascites+Biliary_drainage+Prior_s
urgical_resection+Liver_met+Hospital_type+Tumor_location2+Age_strata+ECO
```

```
G_PS2+T_bil_strata+Alb_strata+CRP_strata+Hb_strata+CA19_9_strata+CCr_

strata+BMI_strata,MI_data,approach='within',method='ps')


result_iptw <-

with(iptw1,coxph(Surv(Time_PFS,Event_PFS)~Treatment),cluster=TRUE)

result_iptw

pool_result<- pool(result_iptw)

summary(pool_result,conf.int=TRUE)
```
