# Supplementary material for: Efficacy of liposomal irinotecan + 5-FU/LV vs. S-1 in gemcitabine-refractory metastatic pancreatic cancer: a real-world study using inverse probability of treatment weighting
Source: J Gastroenterol. 2024 Nov 30;60(3):356–67. doi: 10.1007/s00535-024-02186-9 (PMC11880175; doi:10.1007/s00535-024-02186-9)
Supplement: Supplementary file 4 — Supplementary file4 (DOCX 30 KB) [file 535_2024_2186_MOESM4_ESM.docx]

**Supplemental Table 1. Subsequent treatment**

|  | nal-IRI+5FU/LV | S-1 |
| --- | --- | --- |
|  | (n = 206) | (n = 257) |
| FOLFOX (%) | 51 (24.8) | 9 (3.5) |
| S-1 (%) | 26 (12.6) | – |
| nal-IRI+5FU/LV (%) | – | 21 (8.2) |
| FOLFIRINOX (%) | 8 (3.9) | 2 (0.8) |
| Gemcitabine+nab-paclitaxel (%) | 3 (1.5) | 5 (1.9) |
| Investigational treatment (%) | 2 (1.0) | 2 (0.8) |
| Pembrolizumab (%) | 1 (0.5) | 1 (0.4) |
| Gemcitabine (%) | 1 (0.5) | 1 (0.4) |
| Gemcitabine+S-1 (%) | 1 (0.5) | 0 |
| S-1+irinotecan+oxaliplatin (%) | 1 (0.5) | 0 |
| Surgical resection (%) | 0 | 1 (0.4) |
| Best supportive care (%) | 112 (54.4) | 215 (83.7) |
